# Further optimization of peptide substrate enhanced assay performance for BoNT/A detection by MALDI-TOF mass spectrometry

**DOI:** 10.1007/s00216-017-0421-8

**Published:** 2017-06-02

**Authors:** Dongxia Wang, Jakub Baudys, Kaitlin M. Hoyt, John R. Barr, Suzanne R. Kalb

**Affiliations:** National Center for Environmental Health, Centers for Disease Control and Prevention, 4770 Buford Highway, NE, Atlanta, GA 30341 USA

**Keywords:** Botulinum neurotoxin A, Detection, Mass spectrometry, Peptide substrate, Endopep-MS

## Abstract

Rapid and sensitive detection of botulinum neurotoxins (BoNTs), which cause botulism, is essential in a public health emergency or bioterrorism event. We have previously developed a mass spectrometry (MS)-based functional method, Endopep-MS assay, for the fast detection and differentiation of all BoNT serotypes by affinity enriching the toxin and detecting the serotype-specific cleavage products of peptide substrates derived from the in vivo targets. To improve the performance of the Endopep-MS assay, we report here the further optimization of the peptide substrate for the detection of serotype A botulinum neurotoxins. An increased substrate cleavage was achieved by extending the original peptide N-terminus with optimized amino acid sequence, increasing the detection sensitivity of the method. In addition, the resistance of the substrate to nonspecific hydrolysis was dramatically improved by selectively substituting amino acids at the scissile bond and various other positions of the extended peptide. Moreover, incorporating the N-terminal hydrophobic residues dramatically improved the relative intensity of the cleavage products in the mass spectra. This allowed easy detection of the cleavage products, further enhancing the performance of the assay. The limit of detection for spiked serum sample was enhanced from 0.5 to 0.1 mouseLD_50_ and from 0.5 to 0.2 mouseLD_50_ for spiked stool.

**Graphical abstract:**
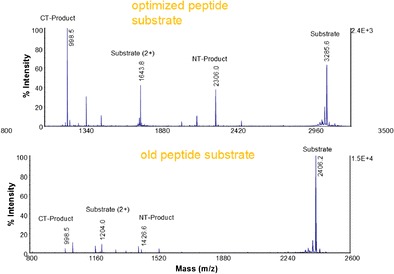
Mass spectra of optimized and old peptide substrates with BoNT/A

Mass spectra of optimized and old peptide substrates with BoNT/A

## Introduction

Botulism is a life-threatening disease occurring in humans and animals caused by intoxication with potent bacterial neurotoxins, known as botulinum neurotoxins (BoNTs) [1–3]. BoNTs are expressed as a single protein of 150 kDa, consisting of two polypeptide chains linked through a disulfide bond. While the heavy chain is responsible for binding and entering target cells, the light chain cleaves at least one of three SNARE proteins in the peripheral neuron, subsequently blocking neurotransmitter release at the neuromuscular junction. BoNTs are classified into seven serotypes (A to G), depending on their antigenic properties and each serotype of BoNT includes several subtypes or variants. Human illness is associated with BoNTs of serotype A, B, E, and F. BoNT/A causes the most severe and longest lasting effects in humans followed by BoNT/B, /F, and /E. Due to their extreme toxicity, the ubiquitous nature of *Clostridium botulinum* spores in the environment, and the ease of preparation, BoNTs are likely agents for bioterrorism [4]. It is essential to have a rapid, simple, and sensitive method for the detection and quantification of botulinum toxins, for timely clinical confirmation of the disease state in botulism.

The mouse bioassay is the oldest method for the detection of active botulinum neurotoxins [5]. Although it is sensitive and robust, the disadvantages of the method include being labor-intensive, time-consuming, requiring at least 4 mL of sample, and use of animals necessitating the development of alternative methods. Alternative methods fall into a limited number of categories; including cell-based assays, immune-based immunological assays, and endoprotease activity-based detection assays [6–8]. Among these alternative detection methods, the measurement of toxin activity offers unique advantages where the method can not only detect the presence of BoNT in samples but also determine whether the toxin is still active or not. Various activity-based assays have been developed by different laboratories. In these methods, the native substrates or their short version mimics are used and the toxin detection is realized by measuring the BoNTs’ cleavage products of the synthetic peptide substrates, using various detection platforms such as HPLC-UV, fluorescence detection, and mass spectrometry [9–13].

A mass spectrometry (MS)-based endopeptidase BoNT activity assay known as Endopep-MS method has been developed in our laboratory [14–17]. The method uses BoNT serotype-specific antibodies to capture toxin in clinical and food samples, incubates the toxin with a peptide that mimics the natural BoNT substrate, and detects the specific cleavage products by high-resolution mass spectrometry. This method allows for the rapid and sensitive detection and differentiation of all seven serotypes of the BoNT using different peptide substrates combined with matrix-assisted laser desorption/ionization (MALDI) mass spectrometry techniques. The method is being implemented in several national and international public health laboratories. During the recently conducted first international proficiency test (EQuATox) for the detection and quantification of botulinum neurotoxins, Endopep-MS proved to be a technique sensitive and selective for the detection, differentiation, and quantification of BoNT in complex matrices [18, 19].

The peptide substrate is one of the key components in the Endopep-MS assay. The sensitivity and selectivity of this in vitro toxin activity measurement method depend directly on the quality of the synthetic peptide mimic. The initial substrate utilized for the detection of type A botulinum neurotoxin in our assay was adapted from a published modified sequence of a special region of the toxin’s native substrate, SNAP-25, that includes a BoNT/A active site [20]**.** The first round of optimization focused on the modification of the peptide termini as well as substitution of the internal amino acid residues of respective synthesized peptides [21]. This optimization achieved a significant improvement in assay sensitivity by enhancing the efficiency of the substrate cleavage and detection of the cleavage products. A high salt washing (2 M NaCl) method has been previously developed in our lab, and the sensitivity of the Endopep-MS assay for stool samples was significantly improved, as a result of the high salt removing non-specific proteases bound to the immuno-capture beads [22]. It is important for this and other in vitro assays to completely reduce or minimize non-specific protease activity because it could alter the substrate such that the toxin cannot cleave it. In addition, non-specific protease activity has the potential to yield false positives. Clinical matrices such as those used to detect BoNT contain a high level of proteases. However, non-specific cleavage of the currently used substrate (Pep-1) was still observed (data not shown) with difficult matrices, especially stool. This prompted us to further optimize the peptide substrate in an attempt to improve assay sensitivity and reduce or diminish the non-specific signals from negative controls for samples in complex matrices.

In this report, we further optimized the BoNT/A peptide substrate to improve the performance of the Endopep-MS assay. By extending the length of the peptide from its N-terminus and optimizing internal amino acid residues within the sequence, a new generation of the peptide substrate was produced which further increased the substrate efficiency, significantly enhanced the visibility of the cleavage products, and dramatically improved substrate resistance toward nonspecific cleavage by proteases other than botulinum neurotoxin, which may be present in clinical sample matrices particularly in stool extract.

## Materials and methods

### Materials

All chemicals were obtained from Sigma-Aldrich (St. Louis, MO), except where indicated otherwise. Fmoc-amino acid derivatives and peptide synthesis reagents were purchased from EMD Chemicals, Inc. (Gibbstown, NJ) or Protein Technologies (Tucson, AZ). The complex forms of the botulinum neurotoxin serotype A were obtained from Metabiologics (Madison, WI). Monoclonal antibodies were provided by Dr. James Marks at the University of California, San Francisco. Streptavidin M-280 Dynabeads were purchased from Invitrogen (Lake Success, NY). Cell culture supernatants of the BoNT/A subtypes A1, A2, A3, A4, A5, A6, and A8 were provided by the National Botulism Surveillance and Reference Laboratory at the CDC or by Dr. Eric Johnson of the University of Wisconsin, and by Dr. Brigitte Dorner of the Robert Koch Institute. Stool and serum were purchased from Zen-bio (Research Triangle Park, NC) and Interstate Blood Bank (Memphis, TN), respectively, and no demographic information was obtained. Because samples were collected without any identifiers or demographic information, these collections are determined to be exempt from human subjects review.

### Peptide synthesis

All peptides were prepared in house by a solid-phase peptide synthesis method using Fmoc chemistry on a Liberty Blue automated microwave peptide synthesizer (CEM, Matthews, NC, USA). Protected peptides were cleaved and deblocked using a reagent mixture of 92.5% trifluroacetic acid (TFA):2.5% water: 2.5% 3, 6-dioxa-1, 8-octanedithiol:2.5% triisopropylsilane and purified on an semi preparative reversed-phase HPLC (Waters 1525 pump system) with a C18 column (XBridge Peptide BEH, 10 mm × 250 mm, 130 Å, 5 μm) using a water:acetonitrile:0.1% TFA gradient. Correct peptide structures were confirmed by MALDI mass spectrometry. All peptides were dissolved in deionized water as a 1 mM stock solution and were stored at −70 °C until further use.

### Endopep-MS toxin activity assay

The in vitro toxin activity assay was carried out as previously described [22]. In brief, the toxin spiked in buffer or biological matrix including serum and stool extract was first immuno-captured by antibodies immobilized on streptavidin beads, followed by an activity assay. After washing steps, the toxin-bound beads were immersed in a 20-μL reaction solution containing 50 μM peptide substrate, 10 μM ZnCl_2_, 1 mg/mL BSA, 10 mM dithiothreitol, and 200 mM HEPES buffer (pH 7.4). The cleavage reaction was conducted at 37 °C for 4 h or other time point indicated in the text.

At the desired time point, 2 μL of the supernatant was transferred from the reaction solution into a PCR tube containing 20 μL of α-cyano-4-hydroxy cinnamic acid (CHCA) at 5 mg/mL in 50% acetonitrile/0.1% TFA/1 mM ammonium citrate and 2 μL of a 1-μM internal standard peptide (IS, 1005.6 Da). Formed cleavage products were measured as the ratio of the isotope cluster areas of the MS peak of the N-terminal product (A_NT_) or C-terminal product (A_CT_) versus an internal standard (A_IS_), or the ratio of A_CT_ versus unreacted intact substrate (A_sub_). The limit of detection was determined as described previously [16].

### Mass spectrometry analysis

Each sample in MALDI matrix was spotted in triplicate on a MALDI plate and analyzed on a 5800 MALDI-TOF instrument (Applied Biosystems, Framingham, MA). The mass spectra of each spot were obtained by scanning from 800 to 5500 *m*/*z* in MS-positive ion reflector mode. The instrument uses an Nd-YAG laser at 355 nm, and each spectrum is an average of 2400 laser shots.

## Results and discussion

### N-terminal extension on the peptide substrate enhanced detection of toxin cleavage product

**Fig. 1:**
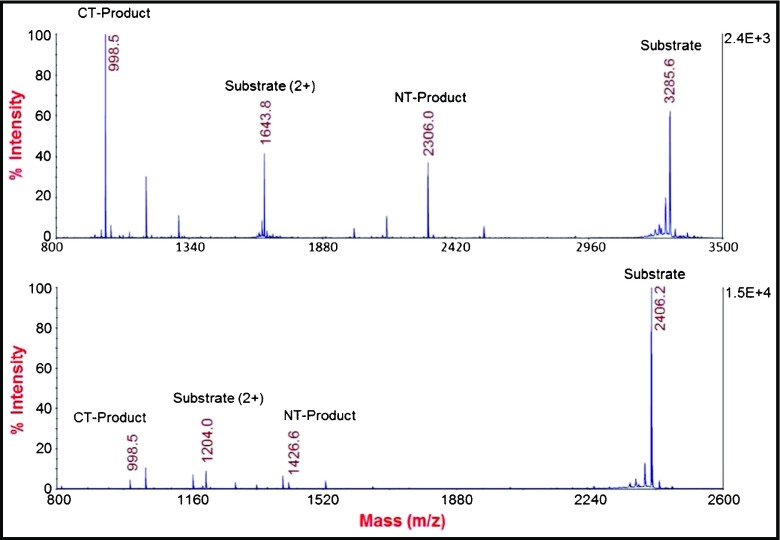
Mass spectra of the cleavage reactions of Pep-18 (*top*) and Pep-1 (*bottom*) by 2 mouseLD_50_ BoNT/A spiked in 0.5 mL PBS/0.05% Tween buffer under identical experimental conditions. Peptide concentration: 100 μM

Since the sequence of the peptide currently used in Endopep-MS method had previously been optimized [21], the further improvement efforts focused on altering the length of this peptide in order to achieve a further improvement. While the initial attempts on shortening the sequence of previous optimized peptide substrate, Pep-1 (Table 1), from either terminus or substituting a few selected internal amino acid residues did not produce the desired result, we changed the strategy to extending the sequence on the N-terminus. This approach was based on two considerations. First, longer peptides are usually more stable and less susceptible to non-specific proteases present in biological matrices presumably due to additional formation of secondary structure in longer peptides. The second consideration lies in improving detection of cleavage products in the mass spectrum. The currently used peptide, Pep-1 (Table 1), is a highly efficient substrate for the BoNT/A enzymatic hydrolysis reaction. However, the intensities of its cleavage products are relatively low, when the amount of toxin present in a sample is low, compared with intensity of unreacted substrate peak due to its high ionization efficiency (Fig. 1 bottom). Although the mass range can be set to exclude the substrate peak, the appearance of all related ions in a single mass spectrum should allow the assay operator to judge the quality of the assay results. Therefore, the research aims were to enhance the relative intensities of the product peaks by modifying the structure of this peptide substrate.

**Table 1:** Relative production of the C-terminal cleavage product (CT product) generated from the hydrolysis of peptide substrates by BoNT/A

Peptide	Sequence	A_CT_/A_IS_	CT product
Pep −1	Ac-RGSNKPKIDAGNQRATRXLGGR-NH_2_	7.30 ± 0.11	100
Pep −2	IDTQNRQIDRIMERGSNKPKIDAGNQRATRXLGGR-NH2	6.63 ± 0.43	91
Pep −3	GNEIDTQNRQIDRIMERGSNKPKIDAGNQRATRXLGGR-NH2	4.65 ± 0.12	64
Pep −4	LDMGNEIDTQNRQIDRIMERGSNKPKIDAGNQRATRXLGGR-NH2	3.69 ± 0.47	51
Pep −5	RHMALDMGNEIDTQNRQIDRIMERGSNKPKIDAGNQRATRXLGGR-NH2	0.51 ± 0.08	7

Underlined letters represent BoNT/A cleavage site

Several new peptides were prepared by adding various numbers of amino acid residues, derived from SNAP25, into the N-terminus of Pep-1, while the C-terminal part of the peptide remained unchanged because the high ionization efficiency of this part ensures the high signal intensity of this cleavage product. It was demonstrated that the addition of 13 extra residues in Pep-2 resulted a slight decrease in the formation of the CT product (Table 1). Further extension by three additional residues (Pep-3) caused about one third reduction in the cleavage product. In addition, the trend of N-terminal elongation resulting in lower cleavage products was observed for Pep-4 and Pep-5, whereas only half of the product was detected from Pep-4 and less than 10% hydrolysis product was observed from Pep-5. This suggests that the addition of more than 13 amino acids to the C-terminus of Pep-1 might disturb its conformation to a significant degree and significantly negatively impact its binding to or cleavage by the toxin.

Using Pep-2 as a new template, we started to manipulate the peptide by substituting or deleting residues in the sequence of the newly attached N-terminal portion. The strategy was applied to examine each individual residue by replacing it with other amino acids or deleting it, starting from the glutamic acid at position 13 (counted from the left). Once a better substitution or deletion is confirmed, it is fixed in the sequence and the process moves to its immediate left amino acid position. Table 2 lists some selected modified peptides that show more CT product detected than the template Pep-2 peptide. When the glutamic acid in the region was substituted with different residues, the peptide (Pep-6) with the alanine substitution achieved approximately 20% improvement in the hydrolysis reaction. Sequentially replacing the residue in the string of “RIM” in Pep-6 to “KAG” in Pep-7 to Pep-9 led to further enhancement. Interestingly, the deletion of isoleucine at the position 8 in Pep-10 did not significantly affect the peptide’s substrate efficiency and removing two residues, R and Q from positions 6 and 7 of Pep-10 even increased the detection of the cleavage product of newly formed peptide, Pep-11. In addition, the substrate activity remained unchanged with additional residue deletion as demonstrated by Pep-12 (removed Q_4_ and N_5_ from Pep-11) and Pep-13 (removed T_3_ from Pep-12). An even more encouraging result was observed when the last three residues “IDD” in Pep-14 were substituted with a string of a very hydrophobic phenylalanine residues. The “FFF” cluster combined with an acetyl-protected N-terminus in Pep-14 yielded a 2.5-fold improvement in product detection.

**Table 2:** CT product yielded from the BoNT/A cleavage of the peptide substrates modified on the amino acid residues in N-terminal part of Pep-2

Pepitde	Sequence	A_CT_/A_IS_	CT product (%)
Pep-2	IDTQNRQIDRIMERGSNKPKIDAGNQRATRXLGGR-NH2	6.63 ± 0.43	100
Pep-6	IDTQNRQIDRIM**A**RGSNKPKIDAGNQRATRXLGGR-NH2	7.95 ± 0.66	120
Pep-7	IDTQNRQIDRI**GA**RGSNKPKIDAGNQRATRXLGGR-NH2	8.12 ± 0.50	122
Pep-8	IDTQNRQIDR**AGA**RGSNKPKIDAGNQRATRXLGGR-NH2	8.63 ± 0.53	130
Pep-9	IDTQNRQID**KAGA**RGSNKPKIDAGNQRATRXLGGR-NH2	9.45 ± 0.40	143
Pep-10	IDTQNRQD**KAGA**RGSNKPKIDAGNQRATRXLGGR-NH2	9.38 ± 1.18	141
Pep-11	IDTQND**KAGA**RGSNKPKIDAGNQRATRXLGGR-NH2	11.70 ± 0.92	176
Pep-12	IDTD**KAGA**RGSNKPKIDAGNQRATRXLGGR-NH2	11.32 ± 0.42	171
Pep-13	IDD**KAGA**RGSNKPKIDAGNQRATRXLGGR-NH2	11.59 ± 0.53	175
Pep-14	**Ac-FFFKAGA**RGSNKPKIDAGNQRATRXLGGR-NH2	16.66 ± 2.00	251

Bold letters represent modified amino acid residues and acetyl (Ac) protected C-terminus

### Improved resistance of the optimized peptide to nonspecific proteases in stool samples

**Fig. 2:**
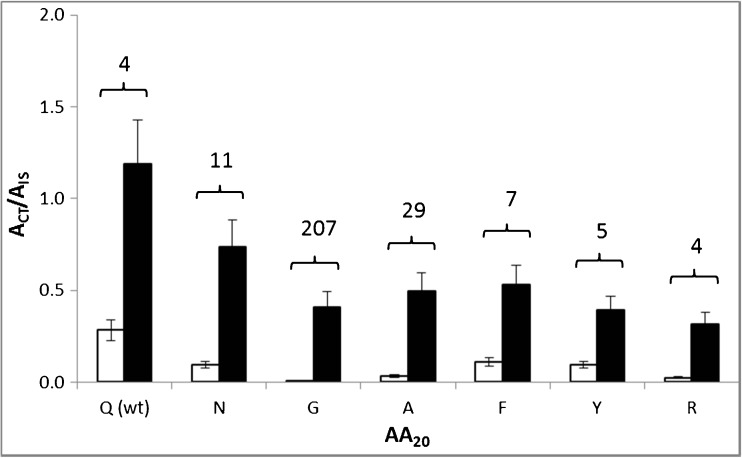
The peak area of the CT product in the mass spectra obtained from the cleavage of the peptide substrates with (■) and without (□) BoNT/A. The toxin of 1 mouse LD_50_ was spiked in 0.1 mL stool extract and captured on antibody-magnetic beads prior to cleavage reaction. The peptides were derived from the Pep-14 (Ac-F_1_F_2_F_3_K_4_A_5_G_6_A_7_R_8_G_9_S_10_N_11_K_12_P_13_K_14_I_15_D_16_A_17_G_18_N_19_Q_20_R_21_A_22_T_23_R_24_X_25_L_26_G_27_G_28_R_29_-NH_2_) by replacing Q_20_ at the cleavage site with some selected amino acid residues. The value on top of each bar pair represents the ratio of the products from the samples with BoNT divided by the ones from the samples without the addition of BoNT toxin. Non-specific cleavage signal originating from impurity and spontaneous degradation of the substrates were subtracted. Reaction condition: 37 °C, 1 h

The resistance of a peptide substrate to endogenous proteases present in complex samples was another parameter to be optimized. It has been demonstrated that the glutamine, one of the two cleavage site residues for BoNT/A substrates, can be replaced by other amino acids without causing significant reduction in the substrate efficiency [21, 23, 24]. To improve the resistance of a peptide substrate toward nonspecific cleavage by stool proteases, we designed several new sequences using Pep-14 as a template by replacing its Q_20_ residue at the BoNT/A cleavage site with several selected amino acids, including the conservative residue of asparagine (N); the hydrophobic residues of glycine (G), alanine (A), phenylalanine (F); and the hydrophilic residues of tyrosine (Y) and arginine (R). As shown in Fig. 2, comparing with the wild-type Pep-14, all other peptides showed reduced nonspecific cleavage product peaks in a negative control (no BoNT/A toxin in the stool extract), suggesting that those substitutions enhanced the resistance of the peptides to hydrolysis by nonspecific protease(s) present in stool samples that are retained on the toxin-antibody-beads complex after the BoNT enrichment step. Although the product formation from the modified peptides was lower than that of template in toxin-containing samples, the ratio of overall product versus nonspecific cleaved product of the three peptides substituted with N, G, and A was significantly higher than that of the wild-type Pep-14. This ratio (207) in glycine-substituted peptide was 50-fold higher than that (4) for Pep-14 due to its very low abundance of the almost undetectable nonspecific cleavage product under the tested experimental conditions; therefore, the peptide (Pep-15) was targeted for further optimization.

In addition to the product peaks, other nonspecific cleaved fragments from the negative control stool samples were observed in the MALDI-TOF mass spectra. The most abundant fragments observed hydrolysis at the peptide bonds of A_5_G_6_ and G_9_S_10_ in Pep-15 (data not shown). These cleavages, though not critical for assay specificity, may consume peptide substrate and consequently negatively impact the sensitivity and quantitative analysis of BoNT/A by the Endopep-MS assay. After testing several peptides with various substitutions at those four positions, two peptides, Pep-16 with P_5_ and Pep-17 with F_10_, were demonstrated to generate reduced fragments cleaved at those two positions, but retained a comparable level of the product peaks in terms of the counts of the CT product, area ratio of product over internal standard, and the ratio of the product versus unreacted substrate (Table 3). For example, no peak corresponding to the CT product of Pep-17 and Pep-18 was detected from the reaction solutions in the negative control stool samples under the experimental condition used, demonstrating the resistance of these two peptides toward nonspecific cleavage at the session bond of the substrates. Additionally, comparable values were observed in terms of the area counts of the cleavage product peak and product/standard ratio from all four peptides in the samples when toxin was spiked in stool extraction, indicating the modifications on these peptides did not lead to significant changes on their substrate efficiency. It is interesting to observe that the area ratio of the CT product peak over the remaining intact substrate peak generated from these modified peptides were different. While Pep-16 and Pep-17 generated either reduced or unchanged ratio upon toxin cleavage, this ratio for Pep-18 was 2-fold higher than the one from the template peptide, Pep-15. More study is needed to understand the underlying mechanism for why the incorporation of two very hydrophobic residues, 2-naphthylalanine, in the N-terminus of the peptide 18 caused a lowered ionization efficiency of the intact peptide. Although the increase in the product/substrate ratio did not necessarily elevate the detection sensitivity of the assay, the significantly increased relative intensity of the peaks of the cleavage product in the mass spectra (Pep-18 vs Pep-1), as shown in Fig. 1, makes these peaks easily visible which should facilitate the recognition of the toxin cleaved product in a quick and clear way. For this reason, we decided to use this peptide as the optimal substrate in future experiments.

**Table 3:** Cleavage of modified peptides in the reactions with or without addition of stool spiked BoNT/A toxin

Pepitde	Sequence^a^	A_CT_ (count)^b^		A_CT_/A_IS_		A_CT_/A_Sub_	
		+ toxin	− toxin	+ toxin	− toxin	+ toxin	− toxin
Pep-15	Ac-FFFKAGARGSNKPKIDAGNGRATRXLGGR-NH2	189,040 ± 34,614	1544 ± 617	5.226 ± 0.261	0.015 ± 0.005	0.953 ± 0.139	0.002 ± 0.002
Pep-16	Ac-FFFKA**P**ARGSNKPKIDAGNGRATRXLGGR-NH2	132,319 ± 8375	1404 ± 90	7.132 ± 0.419	0.027 ± 0.010	0.436 ± 0.035	0.002 ± 0.002
Pep-17	Ac-FFFKAGARG**F**NKPKIDAGNGRATRXLGGR-NH2	177,906 ± 38,945	ND^c^	5.855 ± 0.674	ND	0.881 ± 0.180	ND
Pep-18	Ac-**OOE**KA**P**ARG**F**NKPKIDAGNGRATRXLGGR-NH2	187,233 ± 26,204	ND	4.272 ± 0.374	ND	1.874 ± 0.424	ND

The reactions were conducted in a 20-μL reaction buffer in the presence and absence of BoNT/A toxin at 37 °C for 4 h. The toxin (4 mouseLD50) was spiked in 0.1 mL stool extract and captured on antibody-magnetic beads followed by cleavage reaction

^a^Bold letter represents modified amino acid residue. 0: 2-naphthylalanine

^b^Act A_is_ and A_sub_ represents the peak area CT product (CT), internal standard (IS), and unreacted substrate (Sub), respectively

^c^ND means the CT product was not detected

### Application of the optimal peptide for the detection of BoNT/A subtypes and improvement on assay sensitivity for samples in biological matrices

**Fig. 3:**
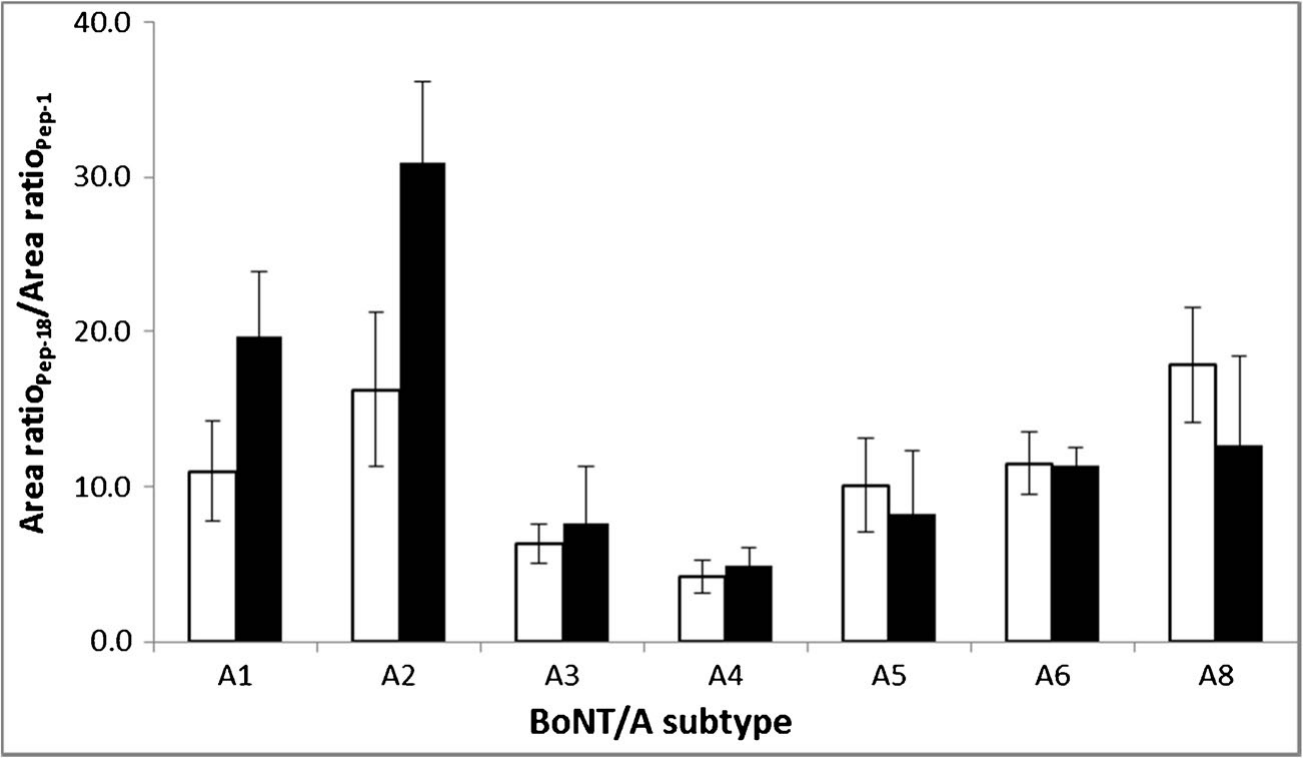
The ratios of the cleavage products, C-terminal (□) or N-terminal (■), from the Pep-18 and Pep-1 hydrolyzed by various BoNT/A subtypes. The area ratio of individual peptides represents the relative intensities of product peak to unreacted substrate peak in mass spectra. The amounts of toxins were known for some of the subtypes but not known for others so that equal amounts of each toxin were not present

To evaluate whether the newly optimized peptide is the suitable substrate for the detection of various subtypes of the type A botulinum neurotoxins, we examined the cleavage of Pep-18 by all so far identified BoNT/A subtypes, from A1 to A8, except A7 which was found in France [25] and was not available to our laboratory. Figure 3 depicts the relative production of the cleavage products, C-terminal and N-terminal, from the Pep-18 versus Pep-1 hydrolyzed by different BoNT/A subtypes. As expected, all subtypes were able to cleave the optimal peptide, Pep-18, with high efficiency, demonstrating the capability of the peptide as a general substrate for various BoNT/A subtypes. In addition, the relative intensities of the product peaks versus the unreacted substrate peaks in mass spectra produced from Pep-18 show significant increases, from about 5-fold for A3 and A4 to 30-fold for A2 reaction, compared with the Pep-1 before optimization. This result further reveals this optimized peptide could serve as a better substrate in the Endopep-MS assay for the sensitive detection of serotype A botulinum neurotoxin and its subtypes.

The performance of this new peptide in terms of assay selectivity and sensitivity was next examined. When Pep-18 was incubated with other serotypes of botulinum neurotoxins including BoNT/B, /C, /D, /E, /F, or /G at high concentrations, no cleavage product was observed in the mass spectra of the cleavage reactions (data not shown), indicating that this peptide was substrate specific to BoNT/A but not any other BoNT serotypes. To evaluate the Endopep-MS assay sensitivity, the specific cleavage of Pep-18 was monitored using the samples with BoNT/A spiked into serum and stool extract, two common clinical sample matrices from botulism patients. Table 4 shows that the limit of detection was determined to be 0.1 and 0.2 mouse LD_50_ for spiked serum and stool, respectively, when Pep-18 was used while the old peptide (Pep-1) produced a 0.5 mouseLD_50_ LOD for both matrices. These results demonstrate the further optimization of peptide substrate significantly improved the sensitivity of the Endopep-MS assay for the detection of type A botulinum neurotoxin. Due to its enhanced performance, this peptide has been included in the CDC developed BoNT test kit, which is currently under FDA review, as a standard test method provided to state health departments for testing of clinical BoNT samples from patients.

**Table 4:** LODs of Endopep-MS assay for BoNT/A spiked in 0.1 mL serum and stool extract

Substrate	LOD (mouseLD_50_/mL)	
	Serum	Stool
Pep-1	0.5	0.5
Pep-18	0.1	0.2

Reaction condition: 37 °C, 4 h

## Conclusion

In this study, we further improved a peptide substrate used for BoNT/A detection in the Endopep-MS assay using a previously optimized substrate, Pep-1, as a template. Through the extension on the N-terminus of the peptide, a significant improvement on detection sensitivity was achieved. In addition, selective substitutions of amino acids at the scission bond and various other positions of the peptide sequence improved its resistance to nonspecific cleavage in biological matrices particularly in stool samples. Moreover, the incorporation of two N-terminal non-natural hydrophobic amino acids dramatically improved the relative intensities of the cleavage products in mass spectra to further enhance the performance of the optimal substrate in the assay. Furthermore, our study demonstrates that this new generation substrate is suitable for use as an efficient reagent for the detection of the subtypes of type A botulinum neurotoxin.
